# Metabolism of cyclic GMP in non-alcoholic fatty liver disease in morbid obesity

**DOI:** 10.1186/1471-2210-11-S1-P73

**Published:** 2011-08-01

**Authors:** Amparo Urios, ML Garcia-Torres, Carla Gimenez-Garzó, Cristina Aguado, Juan A del Olmo, Carmen Marin, Miguel Civera, Fernández N Cassinello, José Ortega, Antonio Ferrandez, J Martinez-Valls, Miguel A Serra, José M Rodrigo, Vicente Felipo, Carmina Montoliu

**Affiliations:** 1 Fundacion Investigacion Hospital Clinico de Valencia. INCLIVA, Spain; 2 Servicios de Hepatologia, Centro de Investigacion Principe Felipe, Valencia, Spain; 3 Laboratory of Neurobiology, Centro de Investigacion Principe Felipe, Valencia, Spain; 4 Endocrinología, Hospital Clinico Universitario, Valencia, Spain; 5 Cirugía, Hospital Clinico Universitario, Valencia, Spain; 6 Anatomia Patologica, Hospital Clinico Universitario, Valencia, Spain

## Aims

To study cGMP metabolism in Nonalcoholic Fatty Liver Disease (NAFLD) in patients with Morbid Obesity (MO).

## Methods

Patients: MO group: 48 patients (40 women and 8 men) diagnosed of MO with an age of 46.9± 7.9. Control group: 19 subjects without overweight and with normal analytical data and normal ultrasound scan of the liver. The following parameters were measured:

A.- Clinical data: Age, sex, weight, height, abdominal and hip perimeters, Body Mass Index (BMI), clinical history and pharmacological treatment, Metabolic Syndrome, morfometry and haematic chemistry, Insulin Resistance (HOMA) and viral markers.

B - Immunomodulators: (Leptin, Adiponectin, CK18Asp396 (CK18), Interleukin-6 (IL-6), aldosterone and rennin).

C - Histological analysis was performed after obtaining hepatic tissue during surgery for MO. The NAFLD Score was calculated: Steatosis Degree, Fibrosis, Inflammation, hepatocyte lesions and other injuries).

## Results

The livers of patients can be divided in 4 groups according to the histological analysis: Normal Liver (2.1 %), Steatosis (60.4 %), Steatohepatitis (35.4 %), Cirrhosis (2.1 %). We find higher values of cGMP in plasma from patients with NAFLD than in the control group (F =2.35; p <0.0001) and in patients with Steatohepatitis (NASH) than in the Steatosis group (F=4.02; p=0.025). Analyzing according to the NAFLD Score, only the fibrosis (F=3.4; p=0.02) shows differences, with no variations according to grade of Steatosis (p=0.05), neither lobular inflammation (p=0.1) nor hepatocellular ballooning (p=0.6).

According to the clinical and analytical data studied, the cGMP level in plasma was higher according to the waist to hip ratio (p<0.05), Metabolic Syndrome (p<0.05): There are no significant differences according to HOMA and BMI. We have not found significant correlations between cGMP and leptin, adiponectin, IL-6, CK-18 or renin, whereas there was a correlation (Pearson=0.43; p=0.14) with aldosterone.

## Conclusion

There is an alteration in cGMP metabolism in NAFLD in patients with morbid obesity. This alteration is associated mainly with development of hepatic fibrosis without alterations related with Steatosis, inflammation or hepatocyte lesions. The alteration in cGMP is independent of the immunomodulators (leptin, adiponectin, CK-18, IL-6, aldosterone and renin) studied.

